# Enhancing the Value Added of Lignin Extracted from *Pinus massoniana* Lamb. via DES Pretreatment

**DOI:** 10.3390/polym18070862

**Published:** 2026-03-31

**Authors:** Hai Cheng, Tong-Qi Yuan, Jinyuan Cheng, Yunni Zhan, Xuelian Zhou, Guigan Fang, Yongjun Deng

**Affiliations:** 1 National Key Laboratory of Forest Food Resource Exploration and Utilization, Research Institute of Forest Products Industry, Chinese Academy of Forestry, Nanjing 210042, China; chenghai@njfu.edu.cn (H.C.); zhanyunni@163.com (Y.Z.); yongjun_deng@126.com (Y.D.); 2 Beijing Key Laboratory of Lignocellulosic Chemistry, Beijing Forestry University, Beijing 100083, China; ytq581234@163.com; 3 Jiangsu Co-Innovation Center of Efficient Processing and Utilization of Forest Resources, International Innovation Center for Forest Chemicals and Materials, College of Light Industry and Food Engineering, College of Chemical Engineering, College of Materials Science and Engineering, Nanjing Forestry University, Nanjing 210037, China; chengjinyuan@njfu.edu.cn; 4 Key Laboratory of Biomass Energy and Material, Jiangsu Province, Key Laboratory of Chemical Engineering of Forest Products, National Forestry and Grassland Administration, Nanjing 210042, China

**Keywords:** diol-based DES, lignocellulose, enzymatic hydrolysis, lignin preservation, lignin-valorization

## Abstract

This study systematically investigated the pretreatment effects of diol-based DESs (deep eutectic solvents) on *Pinus massoniana* Lamb. (*P. massoniana*). A diol-based DES system (Choline chloride (ChCl): AlCl_3_: BDO) was developed to degrade and disassemble *P. massoniana*, thereby facilitating saccharification and achieving the utilization of high-value lignin. The DES-based pretreatment achieved a glucan recovery yield of 92.95% and a xylan yield of 71.73% at 130 °C. Meanwhile, the lignin removal yields reached 61.96% at 130 °C, and the lignin recovered from DES fractionation was also preserved well; moreover, the β-O-4′ linkage content was retained at approximately 51.63%. DES was also demonstrated to be promising for promoting cellulose saccharification, lignin fractionation and enzymatic hydrolysis. The preservation mechanism was speculated to involve the introduction of diol -OH groups at the C_α_-position of the lignin β-O-4′ structure via etherification. In addition, FT-IR indicated that the main structure of cellulose in *P. massoniana* remained unchanged after pretreatment. The grafting of diol onto the C_α_-position of the β-O-4′ linkages was confirmed by 2D-HSQC, which could inhibit lignin further condensation; ^31^P NMR revealed that the total phenolic -OH content increased significantly and was enhanced by pretreatment, which indicated that methoxy and ether bond groups were reduced.

## 1. Introduction

The sustained increase in the consumption of fossil fuel resources over the past decades has brought about an energy crisis and ecological contamination. Lignocellulosic biomass, as one of the most abundant renewable resources on earth, has been recognized as a promising alternative source owing to its abundance and sustainability. It is composed of 35.0–50.0% cellulose, 20.0–35.0% hemicellulose and 10.0–25.0% lignin, which has broad application prospects in the production of fuels and bulk chemicals [1,2,3,4]. Especially in the paper and pulp industry, lignin is typically generated as a by-product and is mostly burned for heat supply, rather than being utilized as a feedstock for high-value chemical production [3,5,6]. Lignin is a natural heterogeneous polyphenolic polymer consisting of three monolignol units: Guaiacyl (G), Syringyl (S), and *p*-hydroxyphenyl (H) units [7,8]. However, its complex structure seriously hinders its high-value utilization, such as in the production of chemical feedstock. Particularly high-content C-O linkages make it extremely depolymerizable [9]. Particularly, Kraft lignin undergoes serious degradation, which seriously hinders its high-value valorization. Condensation reactions are often undergone during the pulping process, which has become a bottleneck constraint in the comprehensive utilization of biomass [10].

The “lignin-first” strategy, which enables the efficient utilization of lignocellulose as a feedstock to obtain lignin, has been proposed as one of the most powerful approaches for generating platform chemicals such as phenol, vanillin, homovanillic acid, and 4-ethylguaiacol [11]. This strategy requires separated lignin with a similar structure compared to native lignin [12]. In contrast, when lignin has highly condensed structures or severely damaged functional groups, this limits its application. However, lignin separation remains challenging due to its inherent heterogeneity, structural complexity, and susceptibility to irreversible degradation and condensation during processing [12]. In recent years, conventional pretreatment technologies such as alkaline solutions, organic solvents, and ionic liquids have been developed for high-quality fractionation, but these methods have not yet addressed these problems. To preserve the native lignin structure and prevent further condensation, Li Shuai et al. [13] developed a formaldehyde stabilization strategy to preserve the original lignin structure during catalytic hydrogenolysis, thereby approaching theoretical monomer yields. However, the application of toxic solvents such as dioxane and formaldehyde in this process poses unavoidable environmental risks. To avoid toxic solvents, alcohol-based pretreatment technologies have been developed and widely applied. Wu et al. [14] put forward a solar energy-actuated ″lignin-first″ approach to maximize the utilization of lignocellulosic biomass, which could obtain as much as 27.0% of aromatic monomers and 88.0% of monosaccharides. Dong et al. [15] found that an ethanol solution pretreatment resulted in the induction of α-etherification of lignin’s side-treated chains. Despite the “lignin-first″ strategy successfully realizing lignin’s conversion into high-value monomers and preserving most of its carbohydrates, these methods are often hindered by side-reactions of the catalyst resulting from inorganics and other impurities in the feedstocks, which reduce the lifecycle of the catalyst [16]. Therefore, developing a new strategy that could extract lignin with a well-preserved structure under mild conditions is imperative.

DESs have emerged as sustainable and renewable solvents for biomass fractionation in recent years, capable of degrading hemicellulose and lignin to enhance cellulose saccharification. EDSs are typically formed by mixting HBA (hydrogen bond acceptor) and HBD (hydrogen bond donor), which coalesce through weak interactions, lowering their lattice energy and resulting in a lower melting point compared with each component individually [17]. Additionally, DESs can be prepared under mild conditions with high purity, homogeneity and monophasic properties [18]. Simultaneously, the previous literature has shown that the glycosidic linkages (C-O-C) in both hemicellulose and lignin–carbohydrate complexes (LCCs) can be cleaved during DES pretreatment [19,20]. Until now, various DES systems have been developed, including organic acid systems (ChCl-lactic, ChCl-OA, and ChCl-formic acid), alcohols (ChCl-EG, ChCl-PG and ChCl-BDO), and lignin-derivative compounds (ChCl-(*p*-HBA), ChCl-(*p*-TSA), and ChCl-guaiacol) [20,21].

The subject under investigation in this study is the development of a diol-based DES system (ChCl: 1, 4-BDO: AlCl_3_) that can efficiently separate and purify lignin from *P. massoniana* while maximizing the preservation of the original structure of the lignin (especially the β-O-4′ linkage, which is the most essential connection in lignin), thereby addressing the critical issue of lignin’s structural degradation during traditional separation and establishing a theoretical and technical groundwork for high valorization of lignin and the advancement of sustainable biomass biorefinery processes. Additionally, to inhibit degradation reactions and modify the lignin structure to obtain the original lignin, we developed a diol-based DES (ChCl:BDO:AlCl_3_) for separating and purifying lignin while preserving the lignin’s primary structure by grafting the -OH of diol onto the lignin sidechains. Heteronuclear single quantum coherence (HSQC) and nuclear magnetic resonance (NMR) were employed to reveal factors that could preserve the β-O-4′ linkages without destroying the lignin structure. These characterization techniques were employed for their high sensitivity and specificity in identifying chemical bonds and functional groups in lignin, offering accurate verification of the structural integrity of lignin after DES pretreatment. By preserving the structural integrity of lignin through DES pretreatment, this study demonstrates a viable strategy to increase the value of the recovered lignin and improve the overall efficiency of biomass biorefineries.

## 2. Experimental Section

### 2.1. Material Preparation

#### 2.1.1. Source of Raw Materials and Chemical Reagents

*P. massionina* was used as the material under investigation and was supplied by a pulp mill in Jiangxi Province, China. It was first processed by a twin-screw extruder (TSE) to reduce its size and then crushed by disk grinding to cause fragmentation. Before pretreatment, the ground materials were dried overnight at 105.0 °C in an iron tray. The chemical composition of raw *PML* comprises rough lignin (31.42%), cellulose (34.79%), hemicellulose (16.03%) and ashes (1.02%). Cellulase (CTe2, Novozymes, 250 PFU/g) and xylanase (X2753, 3500 U/g) were provided by Sigma-Aldrich (Shanghai, China). Choline Chloride (ChCl), (1,4)-Butylene glycol (BDO), AlCl_3_ and acetone were purchased from Sinopharm Chemical Reagent (Shanghai, China).

#### 2.1.2. DES Synthesis, Pretreatment and Lignin Recovery

The DES was synthesized in a 500 mL three-neck flask by mixing ChCl:BDO:AlCl_3_ at a molar ratio of 25:50:1 (optimized based on delignification efficiency). The mixture was heated in a glycerol oil bath at 90 °C ± 2 °C with constant agitation until it formed a homogeneous and transparent liquid. The prepared DES was sealed and placed in a desiccator for further utilization.

The mechanism of forming DES involves BDO providing a hydrogen source and forming a coordination interaction with the hydrogen acceptor of ChCl. In addition, AlCl_3_ strengthens the hydrogen bond network by forming the coordination anion [AlCl_4_]^−^, which enhances the intermolecular interactions within the system. The hydrogen atoms of BDO form hydrogen bonds with lone-pair electrons on ChCl, and then AlCl_3_ endows these with strong electrophilicity and can participate in regulating the distribution of electronic clouds in the mixture. Moreover, Al^3+^ coordinates with oxygen atoms via Al-O bridging (see Supplementary Materials Figure S1) [22,23,24,25,26,27,28].

The raw material pretreatment was conducted with a solid–liquid ratio of 1:10 (100.0 g DES per 10.0 g dry *P. massoniana*) in a 250 mL three-neck flask at various temperatures ranging from 100 to 140 °C for 2 h with constant agitation. After that, the pretreatment was quenched with 300 mL of acetone mixed with water (*v*/*v* = 1:1) and transferred into a 1 L beaker, and then magnetically stirred for 2 h. Next, the mixture was vacuum filtered and then washed with another 200 mL of fresh acetone/water (*v*/*v* = 1:1) twice. The solid was then washed continuously with deionized water until the mixture reached a neutral state, and was then deposited at a low temperature for further utilization. The filtrate was collected separately and rotary-evaporated at 50 °C to remove the residual acetone; after that, 1 L of deionized water was added to regenerate the dissolved lignin. Ultimately, the precipitated lignin was extracted by centrifugation, water-washed three times, and freeze-dried. The dried lignin was collected, sealed, and then deposited in a desiccator away from light for further utilization. After lignin precipitation, the DES was recovered by rotary evaporation at 70 °C to remove excess water, and the recovery yield was calculated by weighing the collected DES.

### 2.2. Materials and Methods

#### 2.2.1. Enzymatic Hydrolysis Evaluation

Enzymatic saccharification efficiency was evaluated in 150 mL flasks using 0.5 g of dry weight sample, to which 1 mL acetate buffer was added to regulate the pH at approximately 4.8, then cellulase (25 FPU/g-glucan) and xylanase (150 U/g-Xylan) were introduced. Deionized water was added to bring the total volume to 20 mL. The sealed flasks were incubated in a shaker at 50 °C and shaken at 150 rpm for 72 h, with tetracycline added to inhibit microbial growth. After enzymatic hydrolysis, 1mL of the sample was collected, centrifuged, and diluted, and the monosaccharide content was determined by High Performance Liquid Chromatography (HPLC).

The enzymatic saccharification yields were calculated by the following equation:

(1) $$\;\mathrm{Enzymatic}\;\mathrm{saccharification}\;\mathrm{yield}\;(\%)\;=\;\frac{\mathrm{glucose}/\mathrm{xylose}\;\mathrm{in}\;\mathrm{the}\;\mathrm{enzymatic}\;\mathrm{hydrolysate}\;(g)}{\mathrm{initial}\;\mathrm{glucose}/\mathrm{xylose}\;\mathrm{in}\;\mathrm{the}\;\mathrm{substrate}\;(g)}\;\times \;100\%\;$$

#### 2.2.2. Chemical Composition Analysis of Raw and Pretreated *P. massoniana*

The chemical composition of the materials was analyzed following the procedure recommended by the National Renewable Energy Laboratory (NREL) [29]. Specifically, 0.3000 g of sample (20–80 meshes) was accurately weighed and hydrolyzed with 3.0 mL of 72 wt% sulfuric acid in a water bath shaker at a constant temperature of 30 °C. Deionized water was then added to dilute the sulfuric acid concentration to 4 wt%, and then the mixture was autoclaved at 120 °C for another 1 h. The monosaccharide content was determined using an Agilent 1260 series HPLC system (Agilent Technologies, Santa Clara, CA, USA) equipped with a Bio-Rad Aminex HPX-87H column from Bio-Rad Laboratories, Inc. (Hercules, CA, USA).

#### 2.2.3. Material Characterization

X-ray diffraction (XRD) analysis was performed on a Bruker Advanced D8 diffractometer (Bruker AXS, Karlsruhe, Germany) equipped with a Cu-K_α_ X-ray source (40 kV voltage, 40 mA current). The test was performed within a 2θ range of 10–40°at 2°/min, and the sample crystallinity index (*CrI*) was calculated based on the following equation:

(2) $$\mathrm{CrI}\;=\;[I_{002}-I_{\mathrm{am}}]/\;I_{002}\;\times \;100\%\;$$

where *I*_002_ represents the maximum diffraction intensity at 22° (corresponding to crystalline cellulose), and *I_am_* represents the minimum diffraction intensity at 18° (corresponding to amorphous regions).

Fourier Transform Infrared Spectrometry (FT-IR) analysis was conducted to investigate the chemical structure changes in *P. massoniana* before and after pretreatment using a Bruker TENSOR 27 spectrometer (Bruker, Mannheim, Germany) in transmittance mode with 32 scans at 4 cm^−1^ resolution over wavenumber ranges of 4000 to 400 cm^−1^.

### 2.3. Preparation and Characterization of Enzymatic Hydrolysis of Material Yield

#### 2.3.1. Preparation of CEL

CEL was separated from raw and pretreated *P. massionanina* samples, which were milled using a QM-QX4 planetary ball mill (Nanjing University Capital Management Co., Ltd., Nanjing, China) for 24 h (duration of validity). The milled samples were subjected to enzymatic hydrolysis to remove carbohydrates at a cellulase dosage of 250 FPU/g of substrate for 48 h. After that, the residual went through the processes of enzymatic hydrolysis for another 48 h. The residue from the enzymatic hydrolysis was then freeze-dried and extracted twice in the dark with aqueous dioxane/water (*v*/*v* = 96/4) for 24 h. The extracts were combined, filtered, and rotary-evaporated to remove the solvent. The resulting CEL was precipitated in deionized water by centrifugation and freeze-dried.

#### 2.3.2. Characterization

The lignin recovery yield was calculated according to the removed lignin after DES pretreatment, and the equation was as follows:

(3) $$\mathrm{Lignin}\;\mathrm{recovery}\;\mathrm{yield}\;(\%)\;=\frac{\mathrm{Lignin}\;\mathrm{recovery}\;\mathrm{from}\;\mathrm{lignin}\text{-}\mathrm{rich}\;(g)}{\;\mathrm{Total}\;\mathrm{removed}\;\mathrm{lignin}\;\mathrm{from}\;\mathrm{the}\;\mathrm{initial}\;P.\;\mathrm{masssoniana}\;}\;\times \;100\%$$

The structural characterization of *P. masssoniana* CEL was analyzed by continuous enzymatic hydrolysis and dioxane extraction, with detailed procedures referred to in a previous publication [30]. Two-dimensional heteronuclear single quantum coherence nuclear magnetic resonance (2D-HSQC) was conducted using a Bruker Ascend^TM^ 600 MHz spectrometer (Bruker, Germany) with the following parameters: spectral width of 166 ppm in the F1 (^13^C) dimension with (256 data points) and 12 ppm in the F2 (^1^H) dimension (1024 data points), *J*_C-H_ coupling constant of 145 Hz, pulse delay of 1.0 s, and 128 scans.

A qualitative and quantitative analysis of the hydroxyl groups (-OH) in the lignin was conducted by ^31^PNMR with a Bruker 600 MHz spectrometer. Specifically, 20 mg of dried lignin was accurately weighed and dissolved in 0.4 mL of anhydrous pyridine-deuterated chloroform solution (*v*/*v* = 1:6). Chromium acetylacetonate (relaxation agent) and cyclohexanol (internal standard) were added, and the mixture was vortexed and mixed until homogenization. A total of 0.1 mL of excess 2-chloro-4,4,5,5-teramethyl-1,3,2-dioxaphospholane (TMDP) was added as phosphitylation reagent, and the mixture was instantly transferred to an NMR tube for analysis.

## 3. Results and Discussion

### 3.1. Reaction Substrate Characterization

#### 3.1.1. Composition Analysis and Enzymatic Monosaccharide Conversion

**Figure 1:**
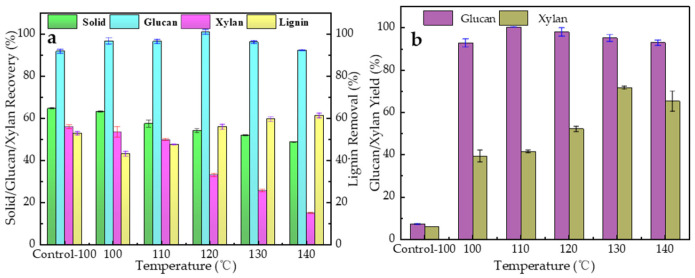
(**a**) Major component recovery yields and lignin removal rate (ChCl:BDO:AlCl_3_ = 25:50:1). (**b**) Enzymatic hydrolysis yield of the polysaccharide (ChCl:BDO:AlCl_3_ = 25:50:1).

As shown in Figure 1a,b, the ChCl/BDO binary system (without AlCl_3_) achieved a solid recovery rate of 64.56%. After pretreatment (Figure 1a), the solid recovery yield from each different temperature intensities were below 65.10% (ranging from 48.79% to 65.10%), which indicated that DES systems could easily adapt to different temperatures. This result suggests that the neutral DES system (ChCl/BDO with a molar ratio of 25:50) exhibited poor lignocellulose fractionation efficiency, likely due to a lack of sufficient Lewis’s acid active sites. With the addition of AlCl_3_ (molar ratio of 25:50:1), the solid recovery rates gradually decreased from 64.56% to 48.87% as the temperature increased from 100 °C to 140 °C, indicating that AlCl_3_ promotes lignocellulose degradation. Compared with the binary system, the ChCl/BDO/AlCl_3_ ternary system significantly reduced the solid recovery rate from 64.59% (100 °C) to 48.87% (140°C), demonstrating enhanced fractionation efficiency [17,20].

Figure 1a,b presents the major component recovery yields after DES pretreatment (ChCl:BDO:AlCl_3_ = 25:50:1, 100–140 °C). As expected, the lignin removal yields increased continuously from 53.07% (100 °C) to 61.52% (140 °C), confirming that AlCl_3_ effectively enhances delignification. The cellulose recovery yields exceeded 90% under severe pretreatment conditions and could eventually reach 100%. The lignin removal rate of the ternary system approached 62% under optimal conditions. This high delignification efficiency may be attributed to the enhanced interaction between ChCl/BDO/DES and lignocellulose, which was facilitated by AlCl_3_-induced modulation of the hydrogen bond network [17].

The cellulose recovery yields gradually increased from 92.94% (100 °C) to 100% (120 °C); however, after that, the cellulose recovery yields slightly decreased from 97.78% (130 °C) to 96.12% (140 °C), which may have been due to a condensation reaction. This result may be attributed to the activation of linkages under high-intensity pretreatment conditions, facilitating cellulose retention in the solid residue [31]. Notably, the hemicellulose recovery rate dropped drastically from 53.65% (100 °C) to 15.25% (140 °C), which was significantly lower than the 56.13% observed at 100 °C in the control group. However, a partial retention of hemicellulose is beneficial for its subsequent utilization, such as in xylose production via enzymatic hydrolysis [32].

#### 3.1.2. Analysis of Raw Pretreated *P. massoniana*

**Figure 2:**
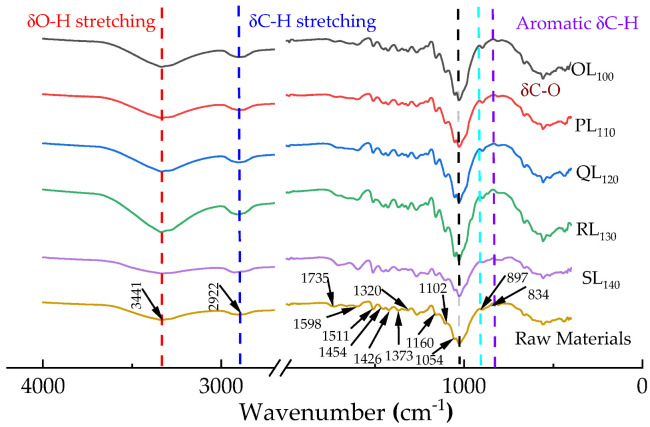
FT-IR spectra of raw materials and pretreated solid samples.

Prior to FT-IR analysis, *P. massionian* samples were dried at 105 °C overnight to achieve absolute dryness. FT-IR spectroscopy was extensively applied to investigate the structural changes before and after pretreatment. As shown in Figure 2, peaks at 896 cm^−1^, 1054 cm^−1^, 1102 cm^−1^ and 1426 cm^−1^ were attributed to C-O-C stretching in amorphous cellulose, C-O vibration in crystalline cellulose, C-O stretching of cellulose, and C-H stretching deformation of cellulose, respectively [33,34,35,36].

A similar trend was observed for C-H stretching in cellulose methylene at 2922 cm^−1^. The peaks at 1598 cm^−1^ (aromatic ring stretching), 1511 cm^−1^ (aromatic ring vibration), 1454 cm^−1^ (C-H deformation of methyl/methylene in lignin) and 834 cm^−1^ (aromatic C-H deformation) were strengthened after the DES pretreatment due to the relative enrichment of lignin in the biomass, while these peaks were significantly weakened after the combinatorial pretreatments, indicating effective lignin removal. Additionally, there were four peaks around 1426, 1373, 1320 and 1160 cm^−1^, which were attributed to C-H deformation, C-H stretching, δC-O vibration of guaiacyl and C-O-C asymmetrical stretching, respectively [37,38]. In contrast, the peak at 1735 cm^−1^ (characteristic of hemicellulose) was almost undetectable, confirming the significant removal of hemicellulose during the DES pretreatment. These results indicate that apparent changes in the lignin structure were observed as the temperature was gradually increased. The intensity of lignin-related peaks weakened after pretreatment, indicating that lignin was removed. However, cellulose- and xylan-related peaks showed a decreasing trend, implying that the dissolution or degradation of cell wall components led to the leaching of lignin and carbohydrates during hydrothermal pretreatment.

#### 3.1.3. XRD Analysis

**Figure 3:**
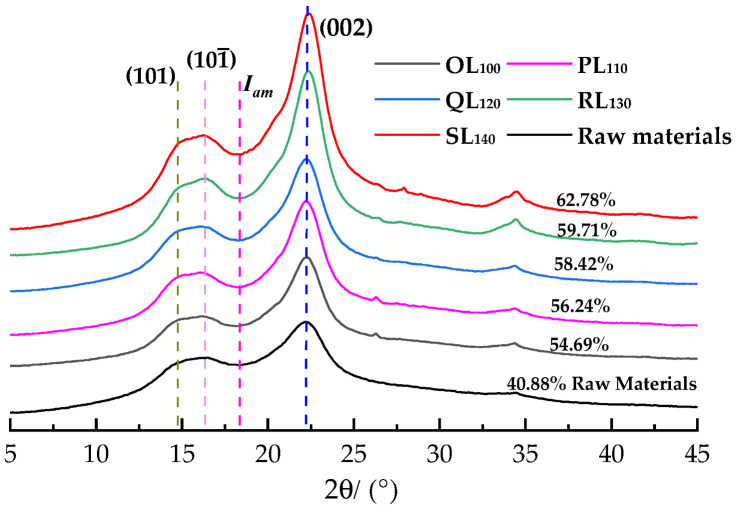
XRD patterns of pretreated materials at different temperatures.

The cellulose crystallinity index (*CrI*) of raw *P. massioniana* was 40.88%, as shown in Figure 3 and calculated by Equation (1). All sample patterns showed similar diffraction peaks, of which distinct peaks indicated apparent absorption near 18° and 22°, corresponding to the crystalline structure of cellulose I. The crystallinity of pretreated samples increased significantly from 40.88% to 54.69%, 56.24%, 58.42%, 59.71% and 62.78% as the temperature increased. This change was mainly attributed to the removal of amorphous lignin and hemicellulose. The result confirmed that the DES system had a positive effect on glucan and xylan removal. *CrI* enhancement was also the result of DES action at specific temperatures [39,40]. Additionally, DES effectively destroyed the amorphous region of cellulose, and the relative crystallinity increased with the removal of lignin and hemicellulose.

#### 3.1.4. Enzymatic Saccharification Analysis

Enzymatic monosaccharide conversion efficiency is a critical indicator for evaluating pretreatment performance. *P. massioniana* only exhibited 7.32% glucan saccharification yield and 6.08% xylan saccharification yield after enzymatic hydrolysis (Figure 1b). Pretreatment with the ChCl/BDO binary system failed to effectively reduce *P. massoniana* recalcitrance. In contrast, the AlCl_3_-enhanced DES system significantly improved the glucan saccharification yields, increasing from 92.95% to 100% when the temperature increased from 100 °C to 120 °C. This efficiency is unexpectedly higher than that of the conventional acid-based DES system, which achieves less than 50% glucan hydrolysis yield. Notably, the glucan yields approached 92.95% at a mild temperature of 100 °C. When the cellulase dosage was reduced to 5 FPU/g-glucan, the saccharification efficiency decreased significantly, indicating that a relatively high cellulase dosage was required to ensure efficient enzymatic hydrolysis. In summary, the AlCl_3_-enhanced DES system achieves satisfactory enzymatic saccharification efficiency while simultaneously preserving cellulose/hemicellulose and removing lignin more effectively than conventional acidic or alkaline DES systems [41,42].

### 3.2. Lignin Characterization

#### 3.2.1. TG and DTG Analysis of Lignin

**Figure 4:**
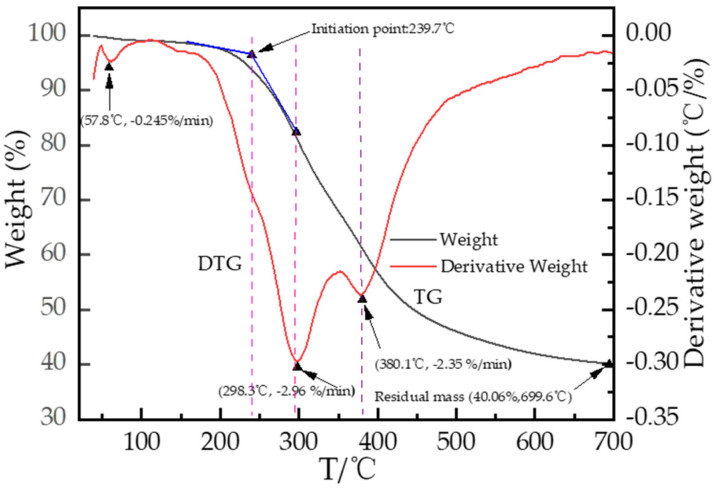
The weight loss curves of TG and DTG of pretreated materials.

The thermodynamic properties of lignin are crucial for its chemical conversion. TG analysis reflects the relationship between sample mass and temperature, while DTG analysis indicates the weight loss rate as a function of temperature variation [43]. As shown in Figure 4, the thermal degradation of lignin samples in this investigation can be divided into four stages: dried, rapid degradation I, rapid degradation II, and slow degradation. Before 293.7 °C in the TG curve, the first weight-loss step mainly involves the β-O-4′ linkage cleavage at 298.3 °C; the thermal degradation rate is mainly related to the carboxylation and dehydrogenation of lignin side chains. From 239.7 to 298.3 °C, the TG curve shows a homogeneous weight-loss trend, which mainly involves the fracturing of Ar-OH, R-OH, β-5′, 4-O-5′ and β-β′ linkages, and partial chemical bonds began to cleave and trigger depolymerization with increases in temperature. In the range of 300 °C to 400 °C, the mass loss rate reaches −2.96%/min to −2.35%/min, and the TG curve shows an accelerated downward trend, indicating significant decomposition of the lignin’s main structure, including carboxylation, dehydrogenation, aromatic ring saturation, and the release of a large number of volatile substances. At temperatures above 400 °C, the TG curve slowly diminishes, indicating that most decomposable substances have been degraded, and the remaining structure exhibits high stability, leading to increased decomposition difficulty. The residual mass approaches 40.06% at 699.6 °C, which is attributed to the pyrolysis and condensation of aromatic rings, consisting mainly of non-decomposable carbonaceous materials and small amounts of inorganic impurities, reflecting the residual proportion after pyrogenic depolymerization [44].

The DTG curve represents the first derivative of weight loss, reflecting the weight loss rate, which indicates the speed of material weight loss. The small peak at 57.8 °C (at which the weight loss rate reaches −0.245%/min) corresponds to the removal of adsorbed water and trace volatile impurities, with a relatively slow mass loss rate. The second stage shows accelerated decomposition at 298.3 °C (weight loss rate reaches −2.96%/min), indicating the rapid degradation of unstable side chain structures and oligomeric components, which is a key stage in lignin pyrolysis [3].

The maximum DTG peak occurs when many chemical bonds in the main structure break, corresponding to the most intense depolymerization reaction and the fastest weight loss rate, which is consistent with the rapid pyrolysis of the main lignin components. TG-DTG analysis reveals that the thermal depolymerization of lignin occurs in multiple stages corresponding to decomposition temperature, suggesting the presence of labile groups in the lignin structure. The two main decomposition peaks are displayed at approximately 298.3 °C and 380.1 °C.

#### 3.2.2. 2D-HSQC NMR Analysis of CEL and Recovered Lignin

**Figure 5:**
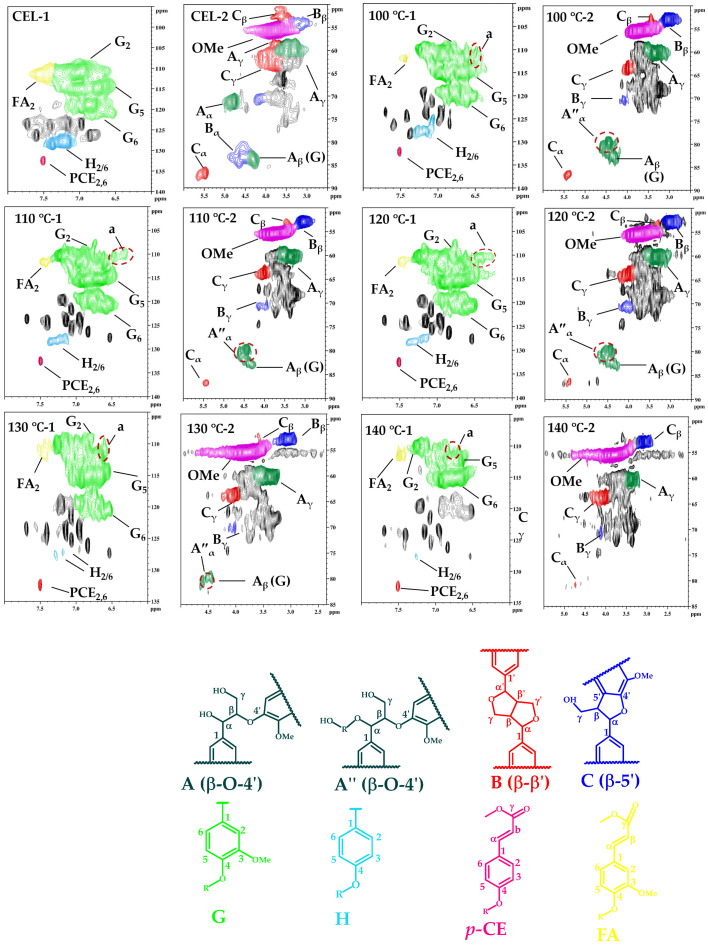
2D-HSQC NMR spectra (aromatic and aliphatic regions) of DES-pretreated PML, identifying major lignin substructures: β-O-4′ aryl ether (A), Cα-grafted β-O-4′ (A″), resinol (B), phenylcoumaran (C), guaiacyl (G), *p*-hydroxyphenyl (H), p-coumarates (*p*-CE), and ferulates (FA). a: a represents condensed lignin content.

The detailed chemical structure variations in residual CEL from the *P. massonina* and DES-pretreated *P. masssonina* were characterized by 2D-HSQC NMR (Ascendtm600-600 MHz, Germany Bruker). The cross-signal assignments of lignin structure are listed in Table 1. CEL was prepared as a control to monitor the structural transformation of lignin functional groups during the pretreatment. The aromatic regions (δ_C_/δ_H_: 160–90/8.0–6.0) and side chain regions (δ_C_/δ_H_: 90–50/6.0–2.5) of the regenerated lignin 2D-HSQC NMR spectra are shown in Figure 5, and the ^13^C-^1^H correlation chemical shifts are provided in Supplementary Table S1.

In aromatic regions (shown in Figure 5), distinct G-unit and H-unit signals were detected in *P. massoniana-CEL*. Additionally, oxidized *p*-coumarates (*p*-CE) and ferulates (*p*-FA) were also distinctly identified. The signal intensity of G_2,5_ in G units gradually weakened and became more less pronounced as the temperature increased [45]. Notably, the β-O-4′ aryl ether (53.67/100Ar) also displayed a prominent cross-signal, possibly due to the high molecular weight of lignin fragments [46]. Strong signals for the methoxy group (-OMe, δ_C_/δ_H_ 55.25/3.72) were also detected in the spectra, implying that lignin molecules contain abundant methoxy group moieties, which are favorable for antioxidant preparation [47]. Interestingly, the content of β-O-4′ aryl ether had obviously disappeared when the pretreatment temperature reached 100 °C, while a new signal peak was observed at δ_C_/δ_H_ 80.59/4.60 in the recovered lignin spectra [46]. The new peak was attributed to β-O-4′ structure derivatives (A″_α_), which were formed by the graft of BDO onto the α-position of the β-O-4′ linkages (Supplementary Figure S2, A-route-1) [18,25,26,27,28,46,48,49,50,51,52,53,54]. As expected, the proposed DES system preserved the β-O-4′ linkage in the regenerated lignin (Table 1). The β-O-4′ bond content in both native and regenerated lignin gradually decreased with rising temperature, recording values of 51.63/100Ar (native), 43.49/100Ar (100 °C), 33.24/100Ar (110 °C), 22.69/100Ar (120 °C), and 17.38/100Ar (130 °C) before disappearing completely at 140 °C (Table 1). During the DES pretreatment, C_α_-OH in an aryl ring structure acted as a leaving group, leading to the hydrolysis of the β-O-4′ linkages and the formation of carbocation intermediates, which typically induce lignin condensation [55]. In this study, BDO acted as a nucleophilic reagent to attack the carbocation intermediates, forming an α-etherified lignin and thereby continuously inhibiting condensation [18,56]. In addition, β-O-4′ linkages and β-β’ (B_β_) and β-5′ (C_β_) linkages also underwent degradation, as evidenced by the weakened signal with enhanced DES pretreatment.

Another result obtained by applying 2D-HSQC NMR is the separation of the signals from the G and H lignin units in the aromatic regions. As illustrated in Figure 4, three prominent peaks at δ_C_/δ_H_ 110.91/6.92, 115.20/6.93 and 119.4.9/6.801 were related to the C_2_-H_2_, C_5_-H_5_ and C_6_-H_6_ correlations of the G units, respectively. Additionally, a minor signal found at δ_C_/δ_H_ 127.6/7.14 corresponded to C_2,6_-H_2,6_ correlations from the H units, which showed characteristics of a G–H-type lignin from softwood. Moreover, compared to CEL, a distinct condensation effect in the regenerated lignin occurred in G units, and the condensation degree apparently increased with elevated temperature. Furthermore, δ_C_/δ_H_ 110.01/7.35 and δ_C_/δ_H_ 132.16/7.51 were attributed to C_2_–H_2_ in ferulates (*p*-FA) and C_2,6_–H_2,6_ in *p*-coumarates (*p*-CE), of which the FA_2_ signal peak gradually weakened until it disappeared completely as the temperature rose [57,58]. This may be attributed to the degradation and fragmentation that occurred of *p*-FA [57,59]. A similar reaction occurred in *p*-CE, which also disappeared.

#### 3.2.3. Quantitative ^31^PNMR Analysis of CEL and Recovered Lignin

**Figure 6:**
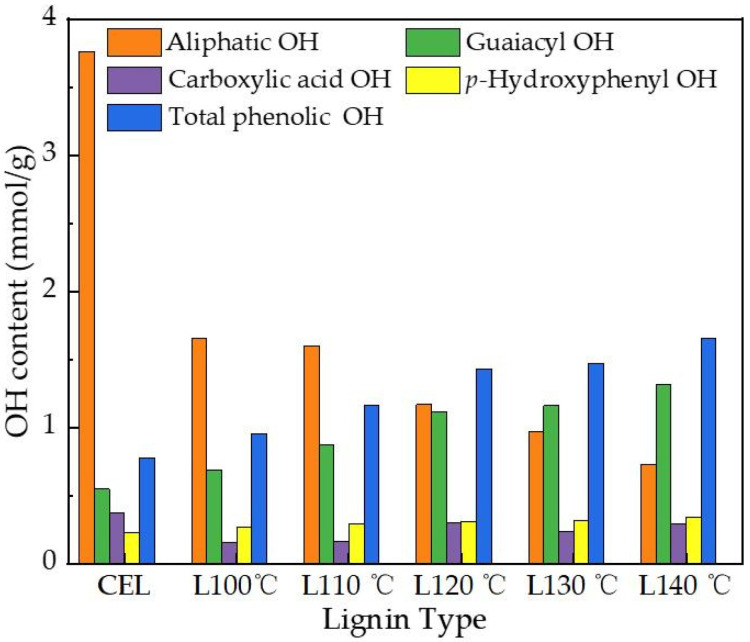
Hydroxyl group contents of CEL and regenerated lignin via ^31^PNMR quantification.

^31^PNMR was employed to investigate the quantitative changes in aliphatic, phenolic, and carboxylic acid OH in lignin before and after DES pretreatment [30]. The total hydroxyl groups exhibited distinct content variations (expressed in mmol/g). As shown in Figure 6, the aliphatic -OH groups of CELs with a content of 3.76 mmol/g, accounting for 74.43% of the total -OH content, were the dominant components. When pretreatment increased from 100 °C to 110 °C, the aliphatic -OH contents decreased slightly, but still accounted for most of the content (with a content of 1.66 mmol/g at 100 °C and 1.59 mmol/g at 110 °C). Compared with *CEL*, the total phenolic -OH contents of pretreated *P. massoniana* showed a mild increase at low temperature, which is consistent with the 2D-HSQC characterization results, confirming the effective preservation of β-O-4′ linkages at 100 °C–110 °C. This mild variation in phenolic -OH at low temperatures was consistent with a previous investigation, which reported that mild temperature conditions (*T* ≤ 100 °C) in an acidic DES pretreatment could retain the main ether linkages of lignin, thereby preventing excessive condensation of phenolic -OH content [59]. However, with further temperature increases, the aliphatic OH contents decreased from 1.16 mmol/g (120 °C) to 0.73 mmol/g (140 °C), which was attributed to the occurrence of condensation reactions in aliphatic -OH during the pretreatment process. The total phenolic -OH content increased from 1.16 mmol/g (100 °C) to 1.47 mmol/g (130 °C) and ultimately reached 1.65 mmol/g (140 °C). Meanwhile, Guaiacyl -OH content also greatly increased from 0.68 mmol/g (100 °C) to 1.31 mmol/g (140 °C). These results indicate that the β-O-4′ bond was partially cracked, leading to a significant increase in the relative content of phenolic -OH compared to aliphatic -OH and suggesting that severe condensation reactions occurred under harsh pretreatment conditions [58]. In addition, an H-unit signal was also demonstrated in CEL via ^31^PNMR, which, coincidentally, corresponded to the results of 2D-HSQC in the diagnosis of H-unit signals. H-unit contents exhibited a slight growth trend that increased from 0.27 mmol/g (100 °C) to 0.31 mmol/g (130 °C) and 0.35 mmol/g (140 °C), which was due to the cleavage of β-O-4′ linkages. With combined 2D-HSQC and ^31^PNMR analysis, the results of the local fragmentation of lignin after DES pretreatment became more remarkable. Due to the β-O-4′ structure’s cleavage, the total phenolic group content showed a relative growth trend compared to aliphatic -OH, which indicated that relevant functional groups underwent more significant condensation under harsh conditions.

#### 3.2.4. Nucleophilic Reagent Efficacy, DES Structure Stability and Related Thermodynamics in DES Pretreatment System

In the ternary DES (ChCl/1,4-BDO/AlCl_3_) system, BDO serves as the primary nucleophilic reagent, which contains two hydroxyl groups that can easily attack electrophilic sites. Cl^−^ (mainly dissociated from Chicle) acts as an auxiliary species, with strong nucleophilic properties, which can assist in breaking the ether bonds in lignin, and even in lignin–carbohydrate complexes (LCCs) [60,61,62]. AlCl_3_ does not serve as a nucleophile but functions as a Lewis acid catalyst to activate the electrophilic sites of lignin, which enhances reactivity between the nucleophiles and lignin [63]. Under low-temperature conditions (100−110 °C), the DES forms a stable homogeneous liquid structure, which is dominated by a hydrogen bond network and the following coordination bonds: (1) (BDO) HO-(CH_2_)_4_−OH⋯Cl^−^, the hydrogen bond dissociation energy of which is 20–25 kJ/mol, which is easily broken at high temperatures; (2) Al^3+^, which coordinates with oxygen atoms in BDO’s hydroxyl groups to form [Al (BDO)_n_]^3+^ (n = 2–3) coordination complexes. When the temperature exceeds 120 °C, the efficacy of nucleophilic reagents (BDO/Cl^−^) undergoes partial failure and significantly decreases, which causes the DES structure to undergo a partial irreversible transformation [64,65,66]. A theoretical thermodynamics calculation makes it clear that the DES hydrogen bond network’s structural stability can be evaluated by the Gibbs free energy of hydrogen bond formation (Δ*G_H__B_*). The *G_H_* can be calculated by the Gibbs–Helmholtz equation, ${\Delta G}_{\mathrm{HB}\;}\;=\;{\Delta H}_{\mathrm{HB}\;}\;-\;T{\Delta S}_{\mathrm{HB}}$, where ${\Delta H}_{\mathrm{HB}\;}$ = −22 kJ/mol represents the enthalpy of hydrogen bond formation; *T* represents the DES system pretreatment temperature; and ${\Delta S}_{HB}=-58\;J/(\mathrm{mol}\;\cdot \;K)$ represents the entropy changes during hydrogen bond formation. The results of this calculation are as follows [67,68,69].

When T = 373 K (100 °C): ${\Delta G}_{\mathrm{HB}\;}=\;-22-373\;\times \;(-0.058)\;=\;-$0.366 kJ/mol < 0, indicating that the hydrogen bond formation is spontaneous, and the DES structure is still stable.

When T = 403 K (130 °C): ${\Delta G}_{\mathrm{HB}\;}=\;-22-403\;\times \;(-0.058)\;=\;1.374\;\mathrm{kJ}/\mathrm{mol}\;$> 0, indicating that the hydrogen bond formation is non-spontaneous, and the hydrogen bond network is destroyed.

The partial structural changes in DES at high temperatures do not lead to complete loss of pretreatment efficacy, but its efficacy is significantly reduced. An increase in temperatures promotes a forward reaction of coordination structure transformation, which weakens the ability of Al^3+^ to activate lignin’s electrophilic sites.

## 4. Conclusions

The study developed a novel pretreatment system employing AlCl_3_ as a Lewis acid reinforcer for *P. massoniana*. The system exhibits exceptional selectivity, achieving over 62% lignin removal while retaining the majority of glucan and xylan. This selectivity fractionation efficiently enables near-theoretical enzymatic saccharification (approaching 100% at 120 °C). Importantly, the recovered lignin retains abundant β-O-4′ linkages, a crucial feature for downstream conversion into aromatic platform chemicals. By integrating efficient fractionation with high-quality lignin recovery, this AlCl_3_-enhanced DES strategy represents a significant advancement towards sustainable and economically viable lignocellulosic biorefineries.

## Figures and Tables

**Table 1:** Quantification of CEL and regenerated lignin (results expressed per 100 Ar units).

Sample	Lignin Removal/%	Lignin Recovery/%	G Units		H	β-O-4′	β″-O-4′	β-β′	β-5′
			Total/%	Condensed/%					
CEL	-	-	90.73	-	9.27	51.63	-	6.31	11.33
L100 °C	43.24	53.07	94.57	9.87	6.02	0	43.49	7.00	7.33
L110 °C	47.74	43.24	95.96	18.55	2.60	0	33.24	5.90	8.31
L120 °C	56.16	47.56	95.77	24.12	5.58	0	22.69	1.69	2.65
L130 °C	59.78	56.16	96.23	30.10	5.39	0	17.38	0.62	2.27
L140 °C	61.52	59.78	96.60	36.10	5.32	0	-	0.43	0.00

## Data Availability

The original contributions presented in this study are included in the article/Supplementary Materials. Further inquiries can be directed to the corresponding authors.

## Supplementary Materials

The following supporting information can be downloaded at: https://www.mdpi.com/article/10.3390/polym18070862/s1, Figure S1: (a) Formation Pathways of Hydrogen Bonds and (b) FT-IR characterizes the Coordination Bonds to validate the structural modulation of the DES system; Figure S2: Pathways of lignin stabilization and condensation reactions in ternary acidity diol-based DES; Table S1: Assignments of ^13^C- ^1^H Cross-Signals in the HSQC NMR Spectra of lignin (CEL).

## References

[1] Kumar A., Jindal M., Maharana S., Thallada B. (2021). Lignin Biorefinery: New Horizons in Catalytic Hydrodeoxygenation to Produce Chemicals. Energy Fuels.

[2] Shen L., Xue X., Wang M., Fan Y., Gu D., Zhu L., Jiang T., Yuan D., Wu H., Wang B. (2023). Review on Catalytic Cracking of Lignin to Produce Fuels and High-Value-Added Chemicals: Advances, Challenges, Opportunities, and Outlook. Energy Fuels.

[3] Cao Y., Zhang C., Tsang D.C.W., Fan J., Clark J.H., Zhang S. (2020). Hydrothermal Liquefaction of Lignin to Aromatic Chemicals: Impact of Lignin Structure. Ind. Eng. Chem. Res..

[4] Zhang B., Li J., Guo L., Chen Z., Li C. (2018). Photothermally promoted cleavage of β-1,4-glycosidic bonds of cellulosic biomass on Ir/HY catalyst under mild conditions. Appl. Catal. B Environ..

[5] Zhang X., Jiang Y., Li W., Zhu L., Wang L. (2024). Production of liquid fuels via catalytic transfer hydrogenation promoting lignin depolymerization on modified Y zeolite: Formic acid as a continuous hydrogen source. Energy Convers. Manag..

[6] Zhang X., Li W., Jiang Y., Zhu L., Wang L. (2024). Valorization of waste lignin: Efficient and steady production of liquid fuels. Ind. Crops Prod..

[7] Da Costa Lopes A.M., Silvestre A.J.D., Coutinho J.A.P. (2023). On the path to improve lignin depolymerization and functionalization into bio-based platform chemicals: A short review. Green Sustain. Chem..

[8] Tian Q., Xu P., Huang D., Wang H., Wang Z., Qin H., He Y., Li R., Yin L., Chen S. (2023). The driving force of biomass value-addition: Selective catalytic depolymerization of lignin to high value chemicals. J. Environ. Chem. Eng..

[9] Su S., Xiao L.P., Chen X., Wang S., Chen X.H., Guo Y., Zhai S.R. (2022). Lignin-First Depolymerization of Lignocellulose into Monophenols over Carbon Nanotube-Supported Ruthenium: Impact of Lignin Sources. ChemSusChem.

[10] Rana M., Taki G., Islam M.N., Agarwal A., Jo Y.-T., Park J.H. (2019). Effects of Temperature and Salt Catalysts on Depolymerization of Kraft lignin to Aromatic Phenolic Compounds. Energy Fuels.

[11] Shen X., Xin Y., Liu H., Han B. (2020). Product-oriented direct cleavage of chemical linkages in lignin. ChemSusChem.

[12] Xu L., Zhang J., Zong Q.-J., Wang L., Xu T., Gong J., Liu Z.-H., Li B.-Z., Yuan Y.-J. (2022). High solid ethylenediamine pretreatment to fractionate new lignin streams from lignocellulosic biomass. Chem. Eng. J..

[13] Shuai L., Amiri M.T., Questell-Santiago Y.M., Héroguel F., Li Y., Kim H., Meilan R., Chapple C., Ralph J., Luterbacher J.S. (2016). Formaldehyde stabilization facilitates lignin monomer production during biomass depolymerization. Science.

[14] Wu X., Fan X., Xie S., Lin J., Cheng J., Zhang Q., Chen L., Wang Y. (2018). Solar energy-driven lignin-first approach to full utilization of lignocellulosic biomass under mild conditions. Nat. Catal..

[15] Dong C., Meng X., Leu S.Y., Xu L., Wu Z., Cravotto G., Fang Z. (2022). Enhancing α-etherification of lignin in Eucalyptus diol pretreatment to improve lignin monomer production. Ind. Crops Prod..

[16] Abu-Omar M.M., Barta K., Beckham G.T., Lautenbacher J.S., Ralph J., Rinaldi R., Román-Leshkov Y., Samec J.S.M., Sels B.F., Wang F. (2021). Guidelines for performing lignin-first biorefining. Energy Environ. Sci..

[17] Xu H., Peng J., Kong Y., Liu Y., Su Z., Li B., Song X., Liu S., Tian W. (2020). Key process parameters for deep eutectic solvents pretreatment of lignocellulosic biomass materials: A review. Bioresour. Technol..

[18] Liu Y., Deak N., Wang Z., Yu H., Hameleers L., Jurak E., Deuss P.J., Barta K. (2021). Tunable and functional deep eutectic solvents for lignocellulose valorization. Nat. Commun..

[19] Xia Q., Liu Y., Meng J., Cheng W., Chen W., Liu S., Liu Y., Li J., Yu H. (2018). Multiple hydrogen bond coordination in three-constituent deep eutectic solvents enhances lignin fractionation from biomass. Green Chem..

[20] Huang C., Zhan Y., Cheng J., Wang J., Meng X., Zhou X., Fang G., Ragauskas A.J. (2021). Facilitating enzymatic hydrolysis with a novel guaiacol-based deep eutectic solvent pretreatment. Bioresour. Technol..

[21] Hong S., Shen X.-J., Xue Z., Sun Z., Yuan T.-Q. (2020). Structure-function relationships of deep eutectic solvents for lignin extraction and chemical transformation. Green Chem..

[22] Harris R.C. (2009). Physical Properties of Alcohol-Based Deep Eutectic Solvents. Ph.D. Thesis.

[23] Chen T., Zhou X., Zhan Y., Liu X., Hung C., Deng Y., Fang G. (2023). Study on rapid dissociation of moso bamboo using ternary low-melting solvents and improvement of enzymatic hydrolysis yield. J. For. Eng..

[24] Chen S., Yu Y., Li Y., Lou Y., Liu Y., Yu H. (2025). Suppressing lignin condensation during deep eutectic solvent pretreatment enhances cellulose accessibility and enzymatic hydrolysis. Green Chem..

[25] Liu H., Chaudhary D., Yusa S.I., Tadé M.O. (2011). Characterization of cellulose nanowhiskers from cotton linter by acid hydrolysis coupled with ultrasonication. Carbohydr. Polym..

[26] Hankins N., Dann S.E., Bruce D.W., Buck M.R., Davis A.P., Fryer J.R. (2014). Amino-functionalised mesoporous silica as a support for phosphotungstic acid: Characterisation and catalytic activity in the oxidation of cyclopentene. Dalton Trans..

[27] Wang S., Liu S., Zhao Y., Wang J., Yu Z. (2019). Insights into the Hydrogen Bond Interactions in Deep Eutectic Solvents Composed of Choline Chloride and Polyol. ACS Sustain. Chem. Eng..

[28] Xuan W., Li H., Wang J., Zhang F., Zhao J., Dai M. (1998). Infrared Spectroscopic Studies of Interactions of the Diols with Aprotic Solvents. Acta Phys.-Chim. Sin..

[29] Sluiter A., Hames B., Ruiz R., Scarlata C., Sluiter J., Templeton D., Crocker D. (2012). Determination of Structural Carbohydrates and Lignin in Biomass—NREL/TP-510-42618, Laboratory Analytical Procedure (LAP).

[30] Huang C., Fang G., Zhou Y., Du X., Yu L., Meng X., Li M., Yoo C.G., Chen B., Zhai S. (2020). Increasing the carbohydrate output of bamboo using a combinatorial pretreatment. ACS Sustain. Chem. Eng..

[31] Kim K.H., Dutta T., Sun J., Simmons B., Singh S. (2018). Biomass pretreatment using deep eutectic solvents from lignin derived phenols. Green Chem..

[32] Zhao J., Yao F., Hu C. (2022). Enhancing enzymatic hydrolysis efficiency of crop straws via tetrahydrofuran/water co-solvent pretreatment. Bioresour. Technol..

[33] Chen Z., Reznicek W.D., Wan C. (2018). Aqueous choline chloride: A novel solvent for switchgrass fractionation and subsequent hemicellulose conversion into furfural. ACS Sustain. Chem. Eng..

[34] Meng X., Sun Q., Kosa M., Huang F., Pu Y., Ragauskas A.J. (2016). Physicochemical structural changes of poplar and switchgrass during biomass pretreatment and enzymatic hydrolysis. ACS Sustain. Chem. Eng..

[35] Xu G., Wang L., Liu J., Wu J. (2013). FTIR and XPS analysis of the changes in bamboo chemical structure decayed by white-rot and brown-rot fungi. Appl. Surf. Sci..

[36] Yoo C.G., Li M., Meng X., Pu Y., Ragauskas A.J. (2017). Effects of organosolv and ammonia pretreatments on lignin properties and its inhibition for enzymatic hydrolysis. Green Chem..

[37] Karimi S., Shekaari H., Halimehjani A.Z., Niakan M. (2021). Solvent-Free Production of 5-Hydroxymethylfurfural from Deep Eutectic Substrate Reaction Mixtures over a Magnetically Recoverable Solid Acid Catalyst. ACS Sustain. Chem. Eng..

[38] Pan Z., Li Y., Wang B., Sun F., Xu F., Zhang X. (2021). Mild fractionation of poplar into reactive lignin via lignin-first strategy and its enhancement on cellulose saccharification. Bioresour. Technol..

[39] Li L., Wu Z., Liang J., Yu L. (2020). Application of deep eutectic solvents in lignocellulosic biomass processing. J. For. Eng..

[40] Yu Y., Li Y., Lou Y., Liu Y., Yu H. (2021). The effect of lignin condensation on cellulase hydrolysis during the dissociation of wood fibers by low melting point solvents. J. For. Eng..

[41] Shen X.-J., Wen J.-L., Mei Q.-Q., Chen X., Sun D., Yuan T.-Q., Sun R.-C. (2019). Facile fractionation of lignocelluloses by biomass-derived deep eutectic solvent (DES) pretreatment for cellulose enzymatic hydrolysis and lignin valorization. Green Chem..

[42] Xu G., Ding J., Han R., Dong J., Ni Y. (2015). Bioresource Technology Enhancing cellulose accessibility of corn stover by deep eutectic solvent pretreatment for butanol fermentation. Bioresour. Technol..

[43] Li M.F., Sun S.N., Xu F., Sun R.C. (2012). Formic acid based organosolv pulping of bamboo (*Phyllostachys acuta*): Comparative characterization of the dissolved lignin’s with milled wood lignin. Chem. Eng. J..

[44] Li C., Zhao X., Wang A., Huber G.W., Zhang T. (2015). Catalytic transformation of lignin for the production of chemicals and fuels. Chem. Rev..

[45] Shen X.-J., Chen T., Wang H.-M., Mei Q., Yue F., Sun S., Wen J.-L., Yuan T.-Q., Sun R.-C. (2020). Structural and morphological transformations of lignin macromolecules during bio-based deep eutectic solvent (DES) pretreatment. ACS Sustain. Chem. Eng..

[46] Dong C., Meng X., Yeung C.S., Tse H.-Y., Ragauskas A.J., Leu S.-Y. (2019). Diol pretreatment to fractionate a reactive lignin in lignocellulosic biomass biorefineries. Green Chem..

[47] Rinaldi R., Jastrzebski R., Clough M.T., Ralph J., Kennema M., Bruijnincx P.C.A., Weckhuysen B.M. (2016). Paving the way for lignin valorization: Recent Advances in Bioengineering, Biorefining and Catalysis. Angew. Chem. Int. Ed. Engl..

[48] Yang S., Qiu Y., Tan G., Ren W., Li X. (2012). Plant Fiber Chemistry.

[49] Deuss P.J., Scott M., Tran F., Westwood N.J., de Vries J.G., Heeres H.J. (2014). Catalytic Conversion of Lignin to Aromatic Chemicals: A Review. ACS Sustain. Chem. Eng..

[50] Xu C., Dolan R.A., Liu C., Zhang Z.C. (2014). Lignin depolymerisation strategies: Towards valuable chemicals and fuels. Chem. Soc. Rev..

[51] Wang H., Tucker M., Ji Y. (2013). Recent development in chemical depolymerization of lignin: A review. J. Appl. Chem..

[52] Sarkanen K., Schuerch C. (1957). Lignin Structure. XI. A Quantitative Study of the Alcoholysis of Lignin. J. Am. Chem. Soc..

[53] Deuss P.J., Scott M., de Vries J.G., Heeres H.J. (2015). Selective Reductive Catalytic Fractionation of Lignocellulosic Biomass. JACS.

[54] Shuai L., Lautenbacher J.S. (2016). Integrating lignin valorization and carbohydrate upgrading via reductive catalytic fractionation. Curr. Opin. Green Sustain. Chem..

[55] Zwilling J.D., Jiang X., Zambrano F., Venditti R.A., Jameel H., Velev O.D., Rojas O.J., Gonzalez R. (2021). Understanding lignin micro-and nanoparticle nucleation and growth in aqueous suspensions by solvent fractionation. Green Chem..

[56] Lan W., Amiri M.T., Hunston C.M., Luterbacher J.S. (2018). Protection group effects during α, γ -Diol lignin stabilization promote high-selectivity monomer production. Angew. Chem. Int. Ed. Engl..

[57] Xie X.-Y., Zhou Q.-Q., Zhu J.-W., Pan W.-W., Zhan Z.-J., Xie B.-G., Zhou W., Xu J.-B. (2023). Progress in Research on Chemical Constituents and Pharmacological Activities of Abies. Chin. Pharm. J..

[58] Cannac M.C., Pasqualini V., Greff S., Fernandez C., Ferrat L. (2007). Characterization of phenolic compounds in *Pinus laricio* needles and their responses to prescribed burnings. Molecules.

[59] Dall’acqua S., Minesso P., Shresta B.B., Comai S., Jha P.K., Gewali M.B., Greco E., Cervellati R., Innocenti G. (2012). Phytochemical and antioxidant-related investigations on bark of Abies spectabilis (D. Don) Spach. from Nepal. Molecules.

[60] Chen S.-X., Liu Y.-Y., Qiu Y.-X., Fang C., Ni J., Wang Z.-Y., Zhang Z.-F. (2022). Advances in the Application of Deep Eutectic Solvent in Biomass Pretreatment. Adv. New Renew. Energy.

[61] Tian J., Lou R., Xue X.Y., Zhang H., Wu S., Xu H. (2021). Pyrolysis Characteristics and Reaction Kinetics Analysis of Nano-Lignin. Chem. Ind. For. Prod..

[62] Zhong L., Wang C., Lyu G.J. (2020). A Review of Deep Eutectic Solvents for Lignin Isolation. Chem. Ind. For. Prod..

[63] Zhang J., Li J., Wang H. (2025). Green Separation of Lignocellulose and Properties of Recombined Composite Materials. J. Chin. Acad. For..

[64] Abbott A.P., Capper G., Davies D.L. (2004). Deep Eutectic Solvents: Green Solvents for the Future. Chem. Rev..

[65] Francisco M., Dominguez A., Jimenez L. (2012). Deep Eutectic Solvents as New Media for Cellulose Dissolution. Green Chem..

[66] Shen Y., Liu Y., Chen S. (2020). Structural Changes of Lignin During Dissolution in Choline Chloride-Lactic Acid Deep Eutectic Solvent. J. Agric. Food Chem..

[67] Zhang Y., Li L., Wang H. (2021). Thermodynamic Analysis of Hydrogen Bond Network in Choline-Based Deep Eutectic Solvents. J. Chem. Thermodyn..

[68] Li J., Zhang H., Chen F. (2022). Coordination Thermodynamics of Metal Ions in Deep Eutectic Solvents. Chem. Phys. Lett..

[69] Wang X., Liu Z., Li P. (2024). Thermodynamic Study on the Stability of ChCl/BDO/AlCl_3_ Deep Eutectic Solvent. J. Chem. Eng. Chin. Univ..

