# Supplementary material for: Enhancing the Value Added of Lignin Extracted from Pinus massoniana Lamb. via DES Pretreatment
Source: Polymers (Basel). 2026 Mar 31;18(7):862. doi: 10.3390/polym18070862 (PMC13074787; doi:10.3390/polym18070862)
Supplement: Supplementary file 1 [file polymers-18-00862-s001.zip › polymers-4167936-supplementary.pdf]

polymers

Supplementary Information

Enhancing the Value Added of Lignin Extracted from *Pinus massoniana* Lamb. via DES Pretreatment

Hai Cheng [1,2,3,4], Tong-Qi Yuan [2], Jinyuan Cheng [3], Yunni Zhan [1,3,4], Xuelian Zhou [1,3,4,*], Guigan Fang [1,3,4,*] and Yongjun Deng [1,3,4]

[1] National Key Laboratory of Forest Food Resource Exploration and Utilization, Research Institute of Forest Products Industry, Chinese Academy of Forestry, Nanjing 210042, China; chenghai@njfu.edu.cn (H.C.); zhanyunni@163.com (Y.Z.); yongjun_deng@126.com (Y.D.)

[2] Beijing Key Laboratory of Lignocellulosic Chemistry, Beijing Forestry University, Beijing 100083, China; ytq581234@163.com

[3] Jiangsu Co-Innovation Center of Efficient Processing and Utilization of Forest Resources, International Innovation Center for Forest Chemicals and Materials, College of Light Industry and Food Engineering, College of Chemical Engineering, College of Materials Science and Engineering, Nanjing Forestry University, Nanjing 210037, China; chengjinyuan@njfu.edu.cn

[4] Key Laboratory of Biomass Energy and Material, Jiangsu Province, Key Laboratory of Chemical Engineering of Forest Products, National Forestry and Grassland Administration, Nanjing 210042, China

* Correspondence: zhouxuelian@icifp.cn (X.Z.); fangguigan@icifp.cn (G.F.)

Table. S1. Assignments of ^{13}C- ^{1}H Cross-Signals in the HSQC NMR Spectra of lignin (CEL).

Lable	δC/δH (ppm)	Assignments
$B_β$	53.37/3.44	$C_β$–$H_β$ in phenylcoumaran
$C_β$	52.50/3.41	$C_β$–$H_β$ in β-β′ resinol
-OMe	55.25/3.72	C–H in methoxyls groups
$A_γ$	60.26/3.21-3.77	$C_γ$–$H_γ$ in β–O–4′ substructures
$B_γ$	63.45/4.01	$C_γ$–$H_γ$ in phenylcoumaran
$C_γ$	70.84/3.94-4.14	$C_γ$–$H_γ$ in β-β
$A_α$	71.16/4.76	$C_α$–$H_α$ in β–O–4′ unit
$A''_α$	80.59/4.60	$C_α$–$H_α$ in β–O–4′ linked to G
$A_β$ (G)	82.65/4.31	$C_β$–$H_β$ in β–O–4′ linked to G
$C_α$	85.0/4.62	$C_α$–$H_α$ in β-β resino
$B_α$	86.6/5.48	$C_α$–$H_α$ in phenylcumaran substructure
G_2	110.91/6.92	$C_α$–$H_α$ in guaiacyl units (G)
G_5	115.20/6.93	C_5–H_5 in guaiacyl units (G)
G_6	119.49/6.81	C_6–H_6 in guaiacyl units (G)
FA_2	110.01/7.35	C_2–H_2 in ferulate (FA)
$H_{2,6}$	127.61/7.14	$C_{2,6}$–$H_{2,6}$ in H units (H)
$PCE_{2,6}$	132.16/7.51	$C_{2,6}$-$H_{2,6}$ in p-coumarate (*p*-CE)

1. Mechanism of DES formation: Hydrogen bonds and Coordination Bonds

The formation of the DES system (ChCl-$AlCl_3$-polyol) relies on intermolecular interactions between the hydrogen bond acceptor (HBA) and hydrogen bond donor (HBD), which collectively disrupt the ordered crystal structure of pure components and reduce the melting point of system. As illustrated in Fig. S1 (Route A), the fundamental framework of the DES is constructed by hydrogen bond interactions between ChCl (acts as HBA) and BDO (acts as HBD) [1].

1.1 Function of ChCl and BDO in Hydrogen Bond Formation

ChCl provides chloride ions ($Cl^−$) with strong electronegativity, which acts as HBA . These Cl^- ions form O-H···$Cl^−$ hydrogen bonds with H atoms from the -OH of BDO (HBD). Significantly, BDO contains two -OH groups at its terminal positions, enabling it to form dual O-H···$Cl^−$ hydrogen bonds with Cl^- ions from different ChCl moleculars. This cross-linking effect constructs a binary supramolecular structure of ChCl, which breaks the regular crystal arrangement of pure ChCl and lowers the melting point of the system [2,3].

1.2 Synergistic Effect of $AlCl_3$ on DES Stabilization

When $AlCl_3$ was introduced into the ChCl-BDO system, it further modulates the intermolecular and enhanced the DES performance:

Coordination bond formation: Al^{3+} ions form Al-O coordination bonds with the oxygen atoms of BDO's-OH groups. This interaction strengthens the electron-withdrawing ability of BDO, thereby improving its HBD capacity and promoting more stable hydrogen bond formation with ChCl.

Ionic strength enhancement: Al^{3+} ions combine with $Cl^−$ ions to form polynuclear ionic cluster ($[AlCl_4]^−$, $[AlCl_7]^−$). These clusters increase the ChCl andonic strength, weaken the lattice energy of ChCl, and synergistically reduce the DES's melting point.

This effect ensures the DES remains in a homogeneous liquid state under mild pretreatment conditions (100-140 ℃), which is critical for efficient lignocellulose deconstruction [3].

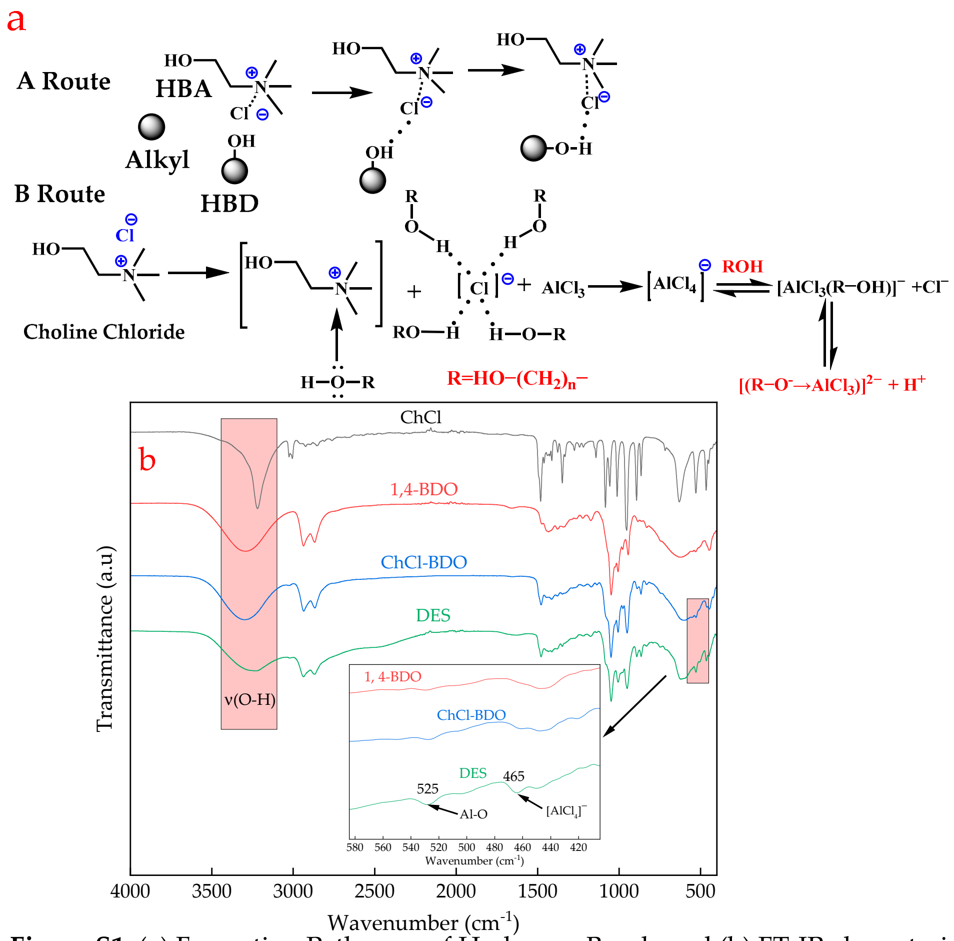

Figure S1. (a) Formation Pathways of Hydrogen Bonds and (b) FT-IR characterizes the Coordination Bonds to validate the structural modulation of the DES system.

1.3 FT-IR characterization the formation mechanism of DES-$AlCl_3$

FT-IR was employed to illustrate the coordination bonds formation mechanisms between ChCl/BDO and AlCl3. The -OH stretching vibration peak of 1,4-BDO at 3400-3200 cm^{-1} was obviously observed. From Fig. S1 (a), it mainly showed that the force attenuation of intermolecular H-bonds in the -OH of ChCl/1,4-BDO was illustrated by the decrease in the hydroxyl stretching frequency in the region of 3250-3150 cm^{-1} [4]. When $AlCl^3$ was added further, hydroxyl peaked at further redshifts to 3100-3000 cm^{-1} with a weak peak and obviously decreased in intensity [4-5]. The slight blue shifts peak at approximately 1470 cm^{-1} was assigned to Al-Cl coordination function, which attributed to the weaken between [Ch]+ and Cl^-, and the vibration environment of the quaternary ammonium group changes, indirectly supporting the participation of in the coordination network [6-7]. The new generated peak of 530-500 cm^{-1} was attributed to Al-O coordination stretching [5].

2. Mechanism of Lignin Condensation Inhibition by Diol-Based DES

The microstructure evolution of lignin during pretreatment (including cleavage of interunit linkages and condensation behavior) is critical for preserving its high-values structure. As in Fig. S2, two distinct pathways dominate lignin transformation: the condensation-prone pathways in acidic environments (Route B) and the condensation-inhibited pathway in diol-based DES (Route A).

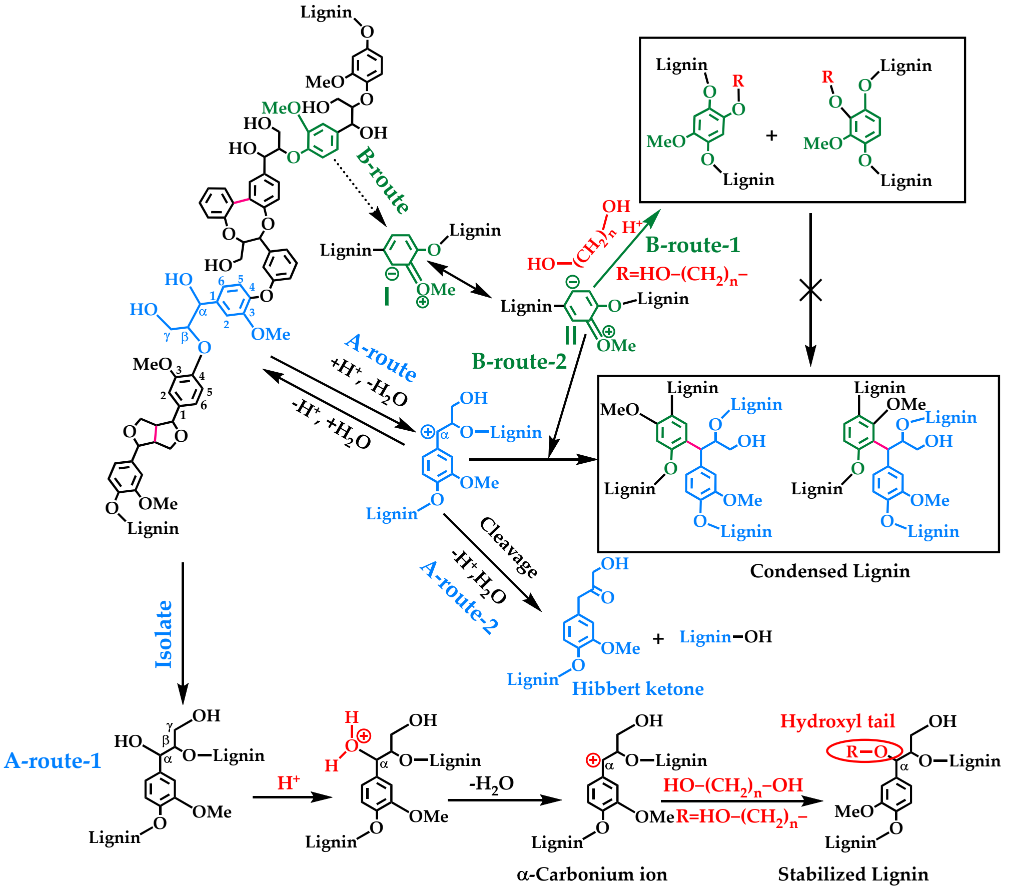

i) $AlCl_3 + R\text{-}OH \rightarrow [AlCl_2(R\text{-}OH)_2]^+ + Cl^-$

ii) $AlCl_3 + R\text{-}OH + Cl^- \rightarrow [(R\text{-}O^-)AlCl_4]^{2-} + H^+$

Figure S2. Pathways of lignin stabilization and condensation reactions in ternary acidity diol-based DES

Lignin extraction interunit condensation from ***P. massoniana*** in an acidic environment (B-route); (A-route) Mechanism of reduced lignin condensation *via* diol-DES based.

2.1 Lignin Condensation in Acidic Environments (Route B)

In conventional acidic pretreatment, lignin undergoes extensive condensation, which degrades its structural integrity and reduces its valorization potential. The mechanism involves two critical steps:

β-O-4 linkage cleavage and oxonium ion formation: Under acidic conditions, the ether oxygen in lignin's β-O-4 linkage (the most abundant interunit linkage,60% of total linkages) reacts with protons (H^+) to form an oxonium ion (tetravalent oxygen salt). This oxonium ion is unstable and undergoes cleavage, generating lignin subunits with reactive benzyl carbocation intermediates (Intermediates Ⅰ and Ⅱ) [4].

C-C condensation via carbocation rearrangement: The benzyl carbocation intermediate (electrophilic) readily reacts with electron-rich aromatic rings in adjacent lignin subunits to form new C-C linkages. Thermodynamic studies have shown that this self-condensation reaction is spontaneous and proceeds at a rate equal to or faster than lignin depolymerization [5]. As a result, technical lignin isolated via acidic pretreatment typically exhibit high condensation degrees (with β-O-4′ linkage content less than 30%) and limited reactivity for monomer production [5-6].

2.2 Lignin Condensation Inhibition by Diol-Based DES (Route A)

The diol-based DES (ChCl-AlC_3-BDO) inhibits lignin condensation through two synergistic mechanisms, preserving the β-O-4′ linkages content (51.63% as detected by HSQC NMR)

2.2.1 Nucleophilic Stabilization of Benzyl Carbocations

BDO (as diol) acts as a nucleophilic reagent to trap reactive benzyl carbocations:

After β-O-4 linkage cleavage, the generated benzyl carbocation (at the C_α position of lignin subunits) reacts with the -OH group of BDO via an S_N2 mechanism. This reaction introduces a hydroxyl-containing side chain (hydroxyl tail) at the C_α-position of the lignin subunit, forming a stabilized lignin derivative (Fig. S2, Route A-1).

The hydroxyl tail at C_α blocks the electrophilic site of the benzyl carbocation, preventing its reaction with adjacent aromatic rings and thus inhibiting C-C condensation. This effect is supported by ^{31}PNMR analysis (main), which shows a significant increase in the content of free -OH groups [7-8].

2.2.2 Suppression of Hibbert's Ketone-Mediated Condensation

Hibbert's ketones (formed *via* β-O-4′ cleavage under acidic conditions) are another key intermediate that promotes lignin condensation. In diol-based DES, BDO can react with Hibbert's ketones to form dioxane-type cyclic structures (Fig. S2, Route A-2). This reaction traps Hibbert's ketones and prevents them from undergoing further condensation reactions [9]. However, in the ChCl-$AlCl_3$-BDO system, this dioxane structure was not detected *via* 2D-HSQC NMR. This is attributed to the steric hindrance of the seven-membered ring structure of the dioxane derivative, which makes it energetically unfavorable under the pretreatment conditions [10].

2.3 Comparison with Diol Organogold Pretreatment

Diol-based DES pretreatment exhibits superior lignin preservation ability by comparing it to traditional diol organogold pretreatment. In diol organosolv, the diol concentration and high temperature lead to partial diol degradation and lignin oxidation. In contrast, the DES system uses BDO as a HBD (at a low molar ratio of ChCl: BDO=1:4) and operates at milder temperatures, reducing diol consumption and lignin oxidation. Enzymatic hydrolysis tests show that the DES-isolated lignin produces higher phenolic monomer yields than diol organogold-isolated lignin [6-7].

References

1. Chen, T. et al. Journal of Forestry Engineering, 2023, 8(5): 86-92
2. R. C. Harris, Physical Properties of Alcohol-Based Deep Eutectic Solvents, Ph. d. Thesis, University of Leicester, 2009.
3. Chen, S. et al. *Green Chemistry*, 2025, 27(8): 2315-2324.
4. H. Liu, D. Chaudhary, S. I. Yusa and M. O. Tadé, Carbohydr. Polym., 2011, 83, 1591–1597.
5. Hankins N, et al. Dalton Transactions, 2014, 43(35): 13245-13254.
6. Wang S Y, et al. ACS Sustainable Chemistry & Engineering, 2019, 7(9): 7760-7767.
7. Dai M. et al. Acta Physiochemical Sinica, 1998, 14(6): 514-519.
8. Yang Shuhui.et al. Plant Fiber Chemistry [M]. China Light Industry Press,2012.
9. Matthew R. et al. ACS Sustainable Chemistry & Engineering 2014 2 (3), 472-485.
10. C. Xu, R.A.D. et al. Chem. Soc. Rev. 43 (2014) 7485-7500.
11. Wang, H. et al. Journal of Applied Chemistry 2013,1–9.
12. K. Sarkanenet al. J. Am. Chem. Soc. 79 (1957) 4203-4209.
13. P. J. Deuss.et al. Journal of the American Chemical Society, 2015,137,7456-7467.
14. Shuai, L.et al. Current Opinion in Green and Sustainable Chemistry 2,59–63.
